# Waist-To-Hip Ratio Predicts Abnormal Overnight Oximetry in Men Independent of Body Mass Index

**DOI:** 10.3389/fcvm.2021.789860

**Published:** 2021-12-15

**Authors:** Joshua M. Bock, Kirk J. Rodysill, Andrew D. Calvin, Soumya Vungarala, Karine R. Sahakyan, Stephen S. Cha, Anna Svatikova, Francisco Lopez-Jimenez, Virend K. Somers

**Affiliations:** ^1^Cardiovascular Medicine, Mayo Clinic, Rochester, MN, United States; ^2^Division of Preventive, Occupational, and Aerospace Medicine, Mayo Clinic, Rochester, MN, United States; ^3^General Internal Medicine, Mayo Clinic, Rochester, MN, United States; ^4^Department of Cardiovascular Medicine, Mayo Clinic Health System, Eau Claire, WI, United States; ^5^Department of Radiology and Radiological Science, Johns Hopkins University School of Medicine, Baltimore, MD, United States; ^6^Biomedical Statistics and Informatics, Mayo Clinic, Rochester, MN, United States; ^7^Nephrology and Hypertension, Mayo Clinic, Rochester, MN, United States

**Keywords:** obstructive sleep apnea, obesity, body fat distribution, oximetry, body mass index

## Abstract

**Background:** Ambulatory overnight oximetry (OXI) has emerged as a cost-effective initial test for sleep disordered breathing. Obesity is closely associated with obstructive sleep apnea (OSA); however, whether body mass index (BMI) or waist-to-hip ratio (WHR) predicts abnormal overnight OXI remains unknown.

**Methods:** We performed a retrospective cross-sectional study of 393 men seen in the Executive Health Program at Mayo Clinic in Rochester, Minnesota who underwent ambulatory overnight OXI ordered by preventive medicine physicians between January 1, 2004 through December 31, 2010. We compared participant/spouse-reported symptoms (sleepiness, snoring), physician indications for OXI (obesity, fatigue), Epworth Sleepiness Scale scores, anthropomorphic measurements (WHR, BMI), and comorbid medical conditions (hypertension, diabetes) with OXI results.

**Results:** 295 of the 393 men who completed OXI had abnormal results. During multivariate analysis, the strongest independent predictor of abnormal OXI for men was WHR (≥1.0, OR = 5.59) followed by BMI (≥30.0 kg/m^2^, OR = 2.75), age (≥55 yrs, OR = 2.06), and the presence of snoring (OR = 1.91, *P* < 0.05 for all). A strong association was observed between WHR and abnormal OXI in obese (BMI ≥ 30.0 kg/m^2^, OR = 6.28) and non-obese (BMI < 29.9 kg/m^2^, OR = 6.42, *P* < 0.01 for both) men. Furthermore, 88 men with abnormal OXI underwent polysomnography with 91% being subsequently diagnosed with OSA.

**Conclusions:** In ambulatory, predominantly middle-aged men undergoing preventive services evaluation many physician indications for OXI were not predictors of abnormal results; however, WHR strongly predicted abnormal OXI in obese and non-obese men. As such, we suggest middle-aged men who snore and have a WHR ≥1.0 should be directly referred to a sleep clinic for polysomnography.

## Introduction

Obstructive sleep apnea (OSA) affects up to 24% of middle-aged adults in the United States (1) and is expected to increase in prevalence proportionally to the obesity epidemic (2, 3). Not only is OSA associated with excessive daytime sleepiness and the complications therein, but also with numerous cardiometabolic disorders, neurocognitive dysfunction, and overall mortality rates (4–6); unfortunately, the majority of OSA cases in the United States have yet been diagnosed (7). In general medical practice, clinicians are challenged to determine which patients should be referred to formal sleep evaluation, namely overnight polysomnography; an expensive assessment requiring specialized expertise in a sleep center. Patient history and physical examination, even in conjunction with published clinical prediction rules, have poor sensitivity and specificity when identifying the presence of OSA (8–10). Ambulatory overnight oximetry (OXI) has emerged as a cost-effective approach to detect OSA or triage patients to sleep centers for further assessment (11). Indeed, nocturnal oxygen desaturation, measured via OXI, is capable of detecting previously undiagnosed sleep disordered breathing in patients referred for surgery (12) and those with heart failure (13). Subsequent studies reported abnormal OXI identified disordered sleep (14) and predicts the incidence of atrial fibrillation (15) in stroke patients. Additionally, data from the Taiwan Bus Driver Cohort Study found more frequent desaturation during sleep predicted future cardiovascular disease, after adjusting for risk factors, in a sample of over 1,000 subjects (16). Despite the utility of OXI to rule out sleep-disordered breathing being known since the 1990's (17), it is underutilized within primary care clinics (18).

While several factors predispose the development of OSA, evidence suggests obesity patterns are particularly influential and may potentially explain why men appear more at risk than women (19, 20). Obesity is most commonly defined by BMI (21), despite its poor reflection of body composition (22), potentially introducing error when estimating risk of OSA (23–25). To this point, central adiposity appears to be more closely related to the presence of OSA as compared to BMI (23, 26). Given that the sensitivity and specificity of OXI for identification of OSA varies by group tested (17, 27–29), uncovering strategies to maximize the utility of OXI paramount; however, it remains unknown if measures of central adiposity predict abnormal OXI. Thus, we sought to examine the clinical and anthropomorphic factors which predict abnormal OXI and the need for subsequent polysomnography. We hypothesized central adiposity, measured via waist-to-hip ratio (WHR), would be superior than BMI when predicting OXI results.

## Materials and Methods

### Study Participants

We conducted a retrospective cross-sectional study of consecutive, ambulatory male patients seen for screening, health care maintenance, and preventive service examinations, not specific for sleep-related symptoms, in the Executive Health Clinic at Mayo Clinic in Rochester, Minnesota, United States, from January 1, 2004 through December 31, 2010. Participant evaluations included history and physical examinations, as well as measures of height, weight, blood pressure, and fat distribution based on WHR. Regarding the latter, single measurements were made of the waist circumference (at the approximate midpoint between the lowest rib and iliac crest during end-expiration) and hip circumference (at the largest point around the buttocks) (30). An *a priori* WHR threshold of ≥1.0 was used to identify individuals at greatest cardiovascular risk based upon previous data (31). This study was approved by the Mayo Clinic Institutional Review Board. Given the higher prevalence of OSA in men as compared to women (32), and the potential impact of these findings on the risk of developing OSA (19, 20), we elected to exclusively study men.

### Sleep History

Participant- or spouse-reported symptoms suggestive of sleep apnea (e.g., snoring, irregular nocturnal breathing) were recorded during each participant's medical record review as were history of diabetes, smoking, and hypertension. All participants completed an extensive health questionnaire (review of symptoms) specifically asking about fatigue, difficulties sleeping (e.g., inability to fall or stay asleep), irregular night-time breathing (e.g., gasping), and snoring along with an Epworth Sleepiness Scale (24) to identify the presence of excessive daytime sleepiness. Indications for OXI (e.g., obesity, sleepiness, snoring, fatigue) were determined based upon these physician notes and surveys.

### Ambulatory Overnight Oximetry

Overnight OXI was completed in the participants' bedroom or hotel room after the participants received detailed verbal and written instructions on the oximeter (PalmSAT 2500, Nonin Medical Inc., Plymouth, MN, United States) which has excellent comparability with direct measurements from arterial blood (33). Participants maintained a sleep diary containing details on bedtime, periods of awakening, and substance use before bedtime in addition to any peculiarities about their night of sleep. Importantly, participants were not using supplemental oxygen or breathing-assistance devices during their OXI assessment.

Data were sampled every 4 s, collected at 75 Hz, stored locally, and then downloaded the following day for offline analysis (18). Variables included recording time, percentage of sleep time spent ≤ 90%SpO_2_ (CT-90) (29), as well as minimum, maximum, and mean SpO_2_. The oxygen desaturation index (ODI) was also calculated by indexing the number of desaturations ≥4%SpO_2_ per hour of recording time. Findings were interpreted by pulmonologists with tests considered normal if mean SpO_2_ ≥90% without frequent oscillatory variations. Abnormal tests indicative of OSA were identified as: hypoventilatory abnormality with desaturation, compatible with sleep-disordered breathing (oscillatory desaturation events), an oxygen desaturation index ≥15 (ODI≥15), and/or a CT-90 ≥1.0 defined as the percentage of recording time patients spent ≥90% (29).

### Polysomnography and Treatment

Executive Health Program patients are, in general, medically stable (e.g., free of acute medical complications) and seen on an annual or semi-annual basis for healthcare maintenance and preventative care. The medical records of participants who underwent OXI were reviewed longitudinally to determine whether they underwent polysomnography or consultation at a sleep center. We determined whether OSA or another sleep diagnosis was made as well as if PAP or another treatment was recommended.

### Data Analysis

Data are reported as mean ± SD, odds ratio (OR) with 95%CIs, or *n* (%) throughout the manuscript. Univariate and multivariate logistic regression analyses were used to identify end point predictors; all data were checked for multicollinearity before proceeding with analysis. We considered the absence or presence of abnormal OXI, ODI ≥15 vs. ODI <15, and CT-90 ≥1.0 vs. CT-90 <1.0 as secondary outcome variables in our analysis. In addition, we developed then evaluated the performance of a predictive model in our database for abnormal OXI. Regression coefficients of the predictive models were transformed into scores (multiplied by 10 and rounded to the nearest integer). Percentage of participants with abnormal OXI data according to the aggregated score in the predictive model was also calculated. All statistical analyses were performed with statistical analysis software version 9.3 (SAS Institute Inc. Cary, NC, United States) with *P* < 0.05 considered statistically significant for all analyses.

## Results

### Patients and Indications for Overnight Oximetry

**Figure 1:**
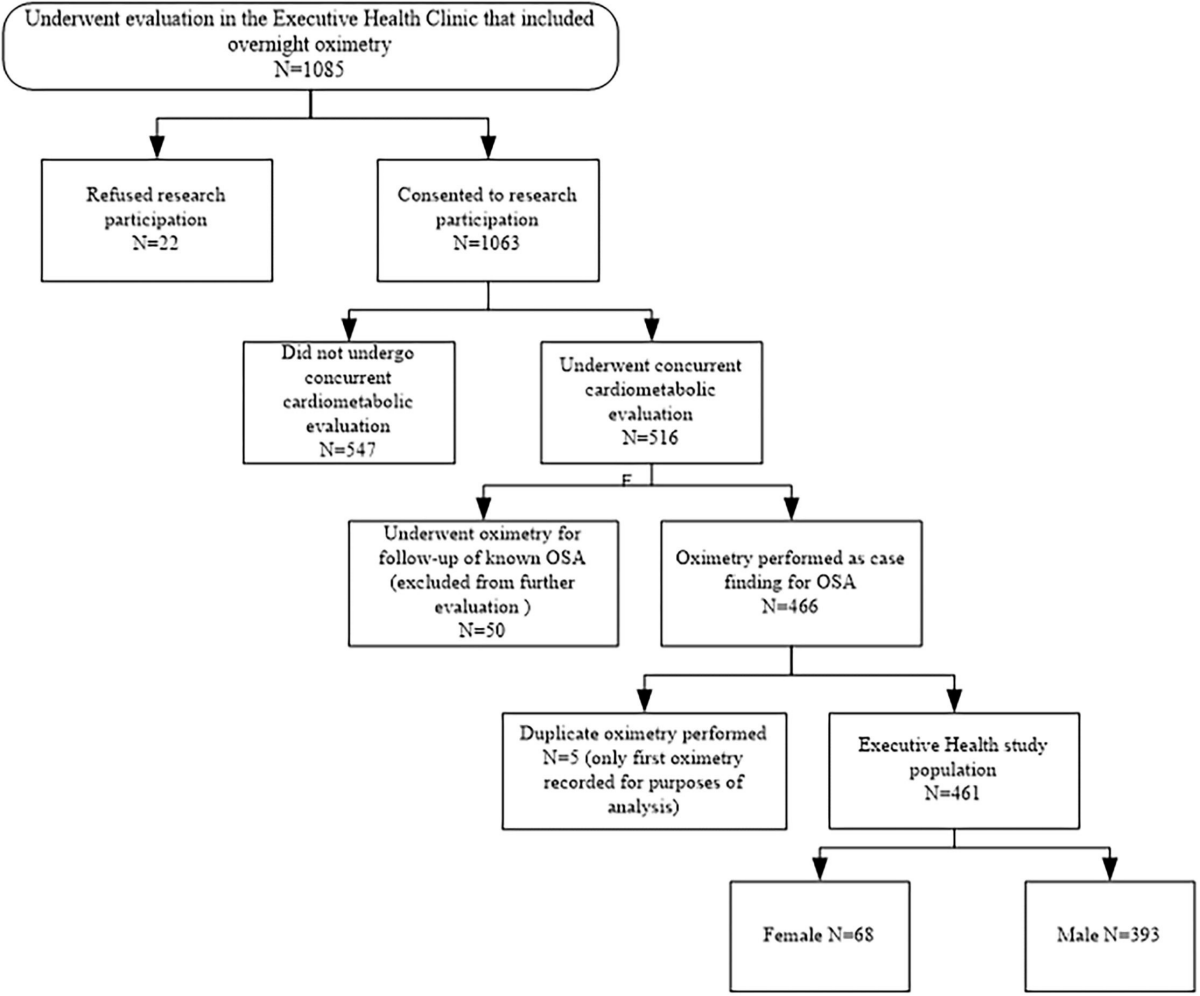
Schematic of patient selection.

A total of 1,085 participants underwent overnight OXI between January 1, 2004 and December 31, 2010; 1,063 consented to medical record review and 516 had concurrent cardiometabolic assessment. Some participants had overnight OXI for follow-up of known OSA (*n* = 50) and were excluded due to use of treatment devices during OXI. In the five participants who had duplicate OXI studies due to artifact during their first recording, only the second artifact-free tests were used. Of the 461 remaining participants, 393 were men and comprised the final study population (Figure 1). Demographical information as well as co-morbidities, prevalence of participant- and spouse-reported symptoms, indications for, and results from, OXI is shown in Table 1.

**Table 1:** Characteristics of study patients.

**Characteristic**	
Age, years	52 ± 9
Weight, kg	99.0 ± 17.7
BMI, kg/m^2^	30.7 ± 4.8
Systolic BP, mmHg	123 ± 16
Diastolic BP, mmHg	80 ± 10
**Comorbid medical conditions, *n* (%)**	
Diabetes mellitus	36 (9)
Hypertension	116 (31)
Smoking	37 (10)
**Patient-reported symptom, *n* (%)**	
Snoring	267 (68)
Fatigue	105 (27)
Sleep difficulties	110 (28)
Irregular breathing	98 (27)
Sleepiness	79 (20)
**Physician indication for oximetry, *n* (%)**	
Sleep difficulties	89 (23)
Sleepiness/fatigue	137 (35)
CV or medical risk	42 (11)
Snoring	319 (81)
Crowded oropharynx	121 (31)
Overweight/obesity	327 (83)
**Sleep evaluation results**	
Epworth Sleepiness Scale score	7 ± 4
Minimum oxygen saturation, %	85 ± 5
4% decreases in oxygen saturation	62 ± 72
CT-90, %	5.4 ± 10.8
ODI	8.1 ± 9.1
Abnormal OXI, *n* (%)	295 (75)
ODI ≥5, *n* (%)	190 (48)
ODI ≥15, *n* (%)	56 (14)
CT-90 ≥1.0, *n* (%)	193 (49)

*Data are presented as mean ± SD or n (%) where indicated. BMI, body mass index; BP, blood pressure; CV, cardiovascular; CT-90, percentage of recording time at oxygen saturation <90%; ODI, oxygen desaturation index; OXI, ambulatory overnight oximetry*.

### Predictors of Abnormal Overnight Oximetry

**Figure 2:**
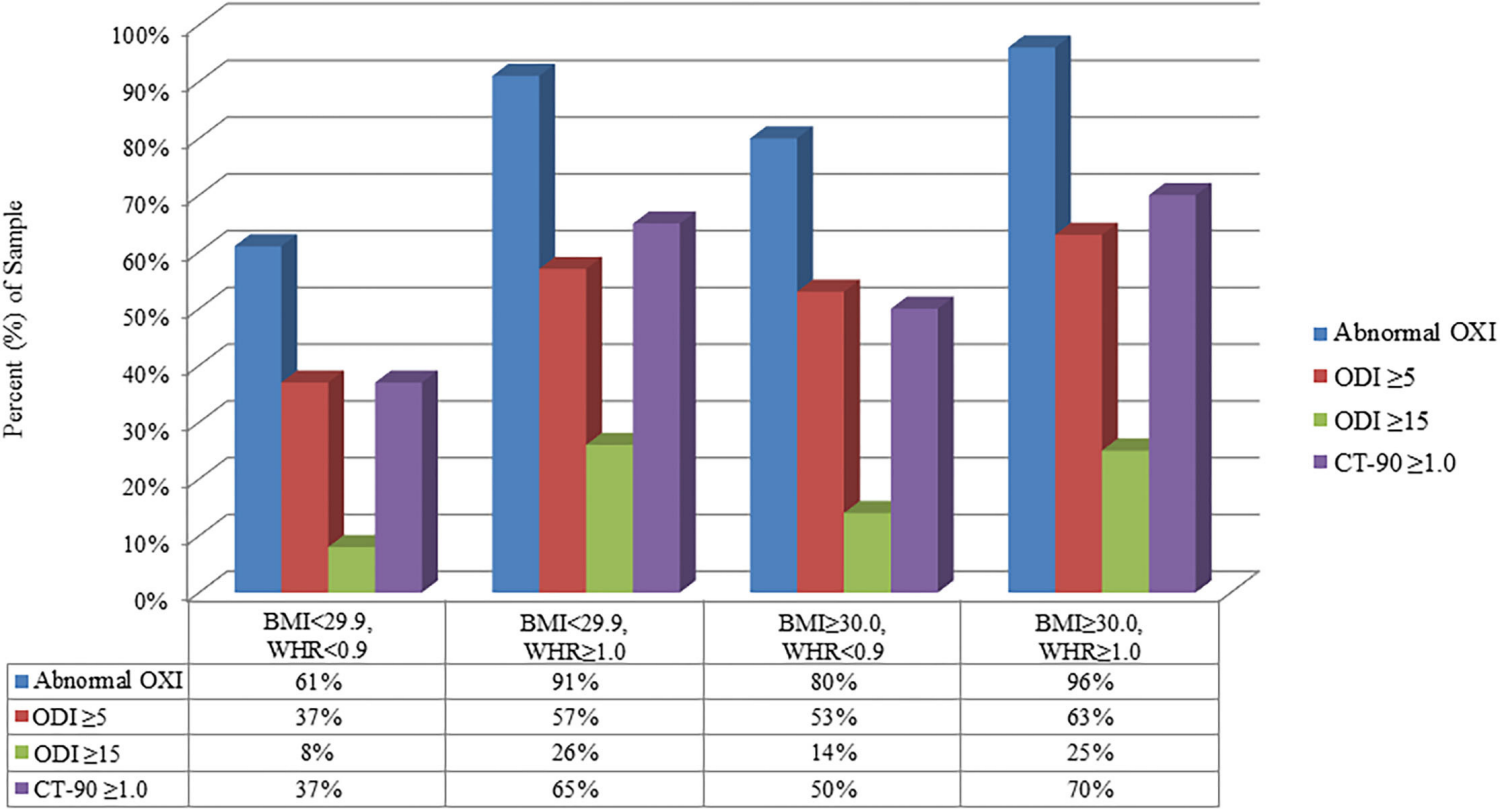
Relative (%) reflection of population with abnormal parameters of ambulatory overnight oximetry (OXI) grouped by BMI and waist-to-hip ratio (WHR). CT-90, percentage of recording time at ODI <90%; ODI, oxygen desaturation index.

Figure 2 shows the prevalence of abnormal OXI, ODI ≥5, ODI ≥15, and CT-90 ≥1.0 categorized by the presence or absence of obesity with and without a WHR ≥1.0. Results from univariate analyses are shown in Table 2. Age, BMI, and WHR ≥1.0 were predictive of abnormal OXI as well as ODI ≥ 15 and CT-90 ≥ 1.0 (*P* < 0.05 for all); however, scores from the Epworth Sleepiness Scale were not (*P* = 0.62, 0.29, and 0.71, respectively). While diabetes mellitus was not indicative of abnormal OXI (*P* = 0.12), it was predictive of both ODI ≥ 15 and CT-90 ≥ 1.0 (*P* < 0.05 for both). Diagnosis of hypertension was predictive of all three indices of abnormal OXI (*P* < 0.01 for all) whereas smoking was not (*P* = 0.07–0.53). Surprisingly, none of the participant-reported symptoms were indicative of abnormal OXI (*P* = 0.06–0.85).

**Table 2:** Univariate analysis of clinical and anthropomorphic variables as predictors of abnormal oximetry.

	**Abnormal OXI**		**ODI** **≥15**		**CT-90** **≥** **1.0**	
	**(*****n*** **=** **295)**		**(*****n*** **=** **56)**		**(*****n*** **=** **193)**	
	**OR (95% CI)**	***P*-Value**	**OR (95% CI)**	***P*-Value**	**OR (95% CI)**	***P*-Value**
Age, years	1.1 (1.0–1.1)	<0.01	1.0 (1.0–1.1)	<0.05	1.0 (1.0–1.1)	<0.01
BMI, kg/m^2^	1.2 (1.1–1.3)	<0.01	1.1 (1.1–1.2)	<0.01	1.1 (1.1–1.2)	<0.01
WHR ≥1.0	1.1 (1.1–1.2)	<0.01	1.1 (1.0–1.1)	<0.01	1.1 (1.1–1.1)	<0.01
ESS	1.0 (1.0–1.1)	0.62	1.0 (1.0–1.1)	0.29	1.0 (1.0–1.0)	0.71
**Comorbid medical conditions**						
Diabetes mellitus	2.2 (0.8–5.8)	0.12	2.6 (1.2–5.7)	<0.05	3.0 (1.4–6.3)	<0.01
Hypertension	2.4 (1.4–4.3)	<0.01	2.8 (1.6–5.0)	<0.01	1.9 (1.2–3.0)	<0.01
Smoking	1.5 (0.6–3.5)	0.38	2.1 (0.9–4.8)	0.07	1.2 (0.6–2.5)	0.53
**Patient-reported symptoms**						
Snoring	1.6 (1.0–2.5)	0.06	1.9 (1.0–3.7)	0.07	1.0 (0.7–1.6)	0.85
Erectile/sexual dysfunction	1.3 (0.7–2.5)	0.40	1.4 (0.7–2.8)	0.35	1.2 (0.7–2.0)	0.57
Fatigue	1.0 (0.6–1.6)	0.83	1.1 (0.6–2.1)	0.74	0.6 (0.4–1.0)	0.05
Sleep difficulties	0.7 (0.4–1.1)	0.09	0.8 (0.4–1.5)	0.39	0.7 (0.5–1.1)	0.11
Irregular breathing	1.5 (0.9–2.7)	0.14	1.2 (0.7–2.3)	0.50	1.5 (0.9–2.3)	0.11
Sleepiness	1.5 (0.6–3.5)	0.38	2.1 (0.9–4.8)	0.07	1.2 (0.6–2.5)	0.53

*BMI, body mass index; WHR, waist to hip ratio; ESS, Epworth Sleepiness Scale score; OXI, ambulatory overnight oximetry; ODI, oxygen desaturation index; CT-90, percentage of recording time at oxygen saturation <90%*.

Findings from multivariate analyses are illustrated in Table 3; WHR ≥ 1.0 was the only measurement that predicted: abnormal OXI, ODI ≥ 15, and CT-90 ≥ 1.0 (*P* < 0.01 for all). Men ages 50–54 years were less likely to have abnormal OXI metrics relative to those ≥55 years (*P* < 0.05 for abnormal OXI and CT-90 ≥ 1.0, *P* = 0.08 for ODI ≥ 15). BMI followed a similar trend as age (≥55 years) was indicative of abnormal OXI and CT-90 ≥ 1.0 (*P* < 0.05 for both) but not ODI ≥ 15 (*P* = 0.21). Diagnoses of diabetes mellitus and hypertension were not indicative of abnormal overnight OXI (*P* = 0.72 and 0.12, respectively); although self-reported snoring was (*P* < 0.05). Participants were differentiated into non-obese (BMI < 29.9 kg/m^2^, *n* = 198) and obese (≥30.0 kg/m^2^, *n* = 195) to further assess the role of obesity in the multivariate models. From these analyses, WHR ≥1.0 was the only variable predictive of abnormal OXI in both obese and non-obese groups (*P* < 0.05 for both). Age ≥ 55 years was indicative of abnormal OXI in obese participants (*P* < 0.05) but this was not observed in their non-obese counterparts (*P* = 0.15). Being 50–54 years (*P* = 0.62 and 0.78) nor having a diagnosis of diabetes mellitus (*P* = 0.56 and 0.89) or hypertension (*P* = 0.22 and 0.42) was predictive of abnormal overnight OXI in either the non-obese or obese cohort, respectively. Similarly, self-reported snoring was not predictive of abnormal overnight OXI in either subgroup (*P* = 0.10 for both).

**Table 3:** Multivariate analysis of clinical and anthropomorphic variables as predictors of abnormal oximetry grouped by body mass index.

	**Abnormal OXI**		**ODI** **≥** **15**		**CT-90** **≥** **1.0**	
	**(*****n*** **=** **295)**		**(*****n*** **=** **56)**		**(*****n*** **=** **193)**	
	**OR (95% CI)**	***P*-Value**	**OR (95% CI)**	***P*-Value**	**OR (95% CI)**	***P*-Value**
WHR ≥1.0	5.3 (2.0–14.2)	<0.01	2.0 (1.0–3.9)	<0.05	2.1 (1.2–3.5)	<0.01
Age 50–54 yrs	1.3 (0.7–2.4)	0.50	1.4 (0.6–3.2)	0.49	1.3 (0.7–2.3)	0.37
Age ≥ 55 yrs	2.1 (1.2–3.9)	<0.05	1.9 (0.9–3.9)	0.08	1.8 (1.1–3.0)	<0.05
BMI ≥ 30 kg/m^2^	2.7 (1.6–4.7)	<0.01	1.5 (0.8–2.9)	0.21	1.7 (1.1–2.6)	<0.05
Diabetes	0.8 (0.3–2.6)	0.72	1.2 (0.5–2.8)	0.77	1.8 (0.8–4.0)	0.18
Hypertension	1.7 (0.9–3.2)	0.12	2.2 (1.1–4.1)	<0.05	1.3 (0.8–2.2)	0.24
Snoring	2.0 (1.1–3.7)	<0.05	2.3 (0.9–5.8)	0.08	1.0 (0.6–1.8)	0.89
**Patients with BMI** ** <29.9 (*****n*** **=** **198)**						
	**Abnormal OXI**		**ODI** **≥** **15**		**CT-90** **≥** **1.0**	
	**(*****n*** **=** **127)**		**(*****n*** **=** **20)**		**(*****n*** **=** **80)**	
WHR ≥1.0	5.0 (1.1–23.7)	<0.05	2.9 (0.9–9.4)	0.09	2.3 (0.9–6.0)	0.10
Age 50–54 yrs	1.2 (0.6–2.7)	0.62	0.6 (0.1–2.9)	0.55	1.2 (0.5–2.7)	0.65
Age ≥ 55 yrs	1.7 (0.8–3.5)	0.15	1.4 (0.5–4.6)	0.55	1.9 (0.9–3.8)	0.08
Diabetes	0.7 (0.2–2.7)	0.56	1.1 (0.2–6.3)	0.93	1.7 (0.5–6.0)	0.44
Hypertension	1.7 (0.7–3.8)	0.22	1.9 (0.7–5.4)	0.25	1.7 (0.8–3.6)	0.14
Snoring	1.9 (0.9–4.2)	0.10	2.0 (0.4–9.8)	0.38	1.1 (0.5–2.5)	0.80
**Patients with BMI** **≥30.0 (*****n*** **=** **195)**						
	**Abnormal OXI**		**ODI** **≥15**		**CT-90** **≥** **1.0**	
	**(*****n*** **=** **168)**		**(*****n*** **=** **36)**		**(*****n*** **=** **113)**	
WHR ≥1.0	5.6 (1.5–20.3)	<0.01	1.7 (0.8–3.8)	0.19	1.9 (1.0–3.6)	<0.05
Age 50–54 yrs	1.2 (0.4–3.9)	0.78	2.0 (0.7–5.9)	0.19	1.5 (0.7–3.5)	0.34
Age ≥ 55 yrs	3.9 (1.2–12.8)	<0.05	2.1 (0.8–5.3)	0.11	1.8 (0.9–3.5)	0.09
Diabetes	1.2 (0.1–10.8)	0.89	1.1 (0.4–3.3)	0.82	2.1 (0.7–6.3)	0.20
Hypertension	1.6 (0.5–4.6)	0.42	2.2 (1.0–5.1)	0.06	1.1 (0.5–2.0)	0.89
Snoring	2.3 (0.9–6.4)	0.10	2.4 (0.8–7.6)	0.14	1.0 (0.5–2.1)	0.95

*BMI, body mass index; WHR, waist to hip ratio; OXI, ambulatory overnight oximetry; ODI, oxygen desaturation index; CT-90, percentage of recording time at oxygen saturation <90%*.

Findings from a stepwise regression analysis are summarized in Table 4. The prevalence of abnormal OXI was 47% for participants with aggregated score <4, 61% for those with the score of 4–8, 67% for those with the score of 9–14 and 89% for study participants with the aggregated score more or equal to 15. Of the variables assessed, WHR ≥1.0 had the highest score (8) followed by BMI ≥30.0 kg/m^2^ (5) and self-reported snoring (4, *P* < 0.05 for all). Both age ≥55 years and hypertension received a score of 3; while the former was predictive of abnormal OXI (*P* < 0.01), the latter did not reach statistical significance (*P* = 0.10).

**Table 4:** Stepwise logistic regression analyses predicting abnormal oximetry.

	**Logistic Regression Coefficient**	**Score**	**OR (95% CI)**	***P*-Value**
Age ≥55 years	0.339	3	2.0 (1.2–3.4)	<0.01
BMI ≥30.0 kg/m^2^	0.485	5	2.6 (1.5–4.6)	<0.01
Hypertension	0.266	3	1.7 (0.9–3.2)	0.10
Snoring	0.360	4	2.1 (1.2–3.8)	<0.05
WHR ≥1.0	0.822	8	5.2 (2.0–13.6)	<0.01

*Data represent the 295 men with abnormal ambulatory overnight oximetry (OXI). Score was obtained by multiplying the logistic regression coefficient by 10 and rounding to the nearest integer. BMI, body mass index; WHR, waist to hip ratio*.

## Discussion

The main findings in this study are that abnormal overnight OXI was associated with WHR (Table 2) independent of obesity (Table 3, Figure 2). That is, WHR predicted abnormal OXI in both participants with (≥30.0 kg/m^2^) and without (<30.0 kg/m^2^) a BMI classification of obese. Moreover, 88 of the 295 men with abnormal overnight OXI underwent follow up polysomnography at Mayo Clinic with 80 (91%) being subsequently diagnosed with OSA. Interestingly, most participant- and spouse-reported symptoms, including sleepiness as defined by the Epworth Sleepiness Scale, were not predictive of abnormal OXI (Table 3). Collectively, these data indicate elevated WHR measurements, particularly in patients who snore, may serve as a robust indication for overnight OXI and could be a cost-effective approach for triaging patients to sleep clinics.

Classically, overnight polysomnography has been the mainstay assessment for identification of sleep-disordered breathing; however, significant costs, prolonged wait times, as well as the requirement of specialized personnel and equipment restrict the volume of patients sleep centers can encounter which may have contributed to the underdiagnosis of OSA in recent years (34). In-home sleep testing is slightly more cost-effective than polysomnography (35) and is not subject to the same wait times, although HSATs are not without limitation. For instance, their data are considered less accurate and less robust (e.g., fewer variables) than polysomnography and subsequently, their cost effectiveness has been subject to scrutiny (36). Along these lines, OXI has minimal associated cost due to its technical simplicity and relatively easy to interpret data. Indeed, recent work from Ayache and Strohl (37) found high inter-clinician reliability in concert with excellent sensitivity and specificity when using a template interpreting OXI. While the amount of data captured via OXI is a fraction of those from polysomnography or HSATs, the clinical utility of nocturnal desaturation is well-studied. That is, we have previously reported hypoxemia independently predicted poor prognosis in post-infarct patients (38) with similar findings reported from other laboratories studying clinical (16, 39) and healthy (40) populations. To our knowledge, the present study is the largest to examine the use of OXI in an unselected, non-referred outpatient population of generally healthy men being seen for screening and health care maintenance examinations.

In the present study WHR, an index of central adiposity, was the best predictor of abnormal OXI among variables included in the multivariate analysis confirming prior studies which reported a positive association between adiposity (41, 42), as well as body fat distribution (43), and risk of OSA. Our findings are particularly novel as hallmark phenotypes of OSA, such as snoring and obese BMI, only had 40–50% the predictive capacity of a WHR ≥1.0 (Table 3). Interestingly, when participants were partitioned into normal and obese BMI cohorts, WHR remained the strongest predictor of abnormal overnight OXI (OR ≥5.0). We recognize participants with an obese BMI (≥30 kg/m^2^) likely have a WHR ≥1.0 which may influence conclusions from the present study. This notion is illustrated in Figure 2 where 96% of participants who met both criteria had abnormal OXI; however, 91% of non-obese participants (<30 kg/m^2^) with a WHR ≥1.0 also had abnormal results. Nevertheless, data from our stepwise regression analysis suggest a synergistic relationship between high BMI and WHR (Table 4). Here, participants with a WHR ≥1.0 in concert with any other variable (e.g., hypertension, snoring) had ≥89% chance of abnormal OXI. In sum, our data indicate traditional signs of OSA (e.g., snoring, obese BMI) have predictive value albeit not as robust as a WHR ≥1.0.

While investigating the mechanism(s) linking central obesity (WHR) and nocturnal oxygen levels is outside the scope of this study, a simple explanation could be disrupted breathing mechanics. That is, greater abdominal obesity may impede tidal volume beyond the compensatory ability of respiratory rate resulting in hypoventilation and subsequently lower SpO_2_. Interestingly, the relationship between central adiposity, specifically visceral fat, and nocturnal oxygen levels appears to be bidirectional as patients with OSA treated with PAP (i.e., improved nocturnal SpO_2_) may demonstrate a reduction in visceral fat (44) although the pathway(s) mediating these observations remain unclear. A potential modulatory role of advancing age on the WHR-OXI should also be mentioned as, in univariate analysis, age predicted abnormal OXI (Table 2) as did being ≥55 yrs with an obese BMI (Table 3). Indeed, aging is accompanied by deleterious changes within bioenergetics and neuromuscular tone of the diaphragm (45) which may promote nocturnal desaturation independent of central adiposity, although an age-adipose interaction cannot be ignored.

### Clinical Implications

These findings are of immediate clinical usefulness as participant-reported symptoms, other than snoring, lack predictive utility for OSA (Table 3). Fortunately, WHR is relatively easy to calculate, interpret (e.g., 1.0 threshold), and technically simple to perform. We used a stepwise logistic regression analysis to develop a score to predict pulmonologist interpretation of OXI as normal or abnormal in male patients (Table 4). As an example, a 56-year-old man who snores and has a WHR ≥1.0 has an 89% chance for abnormal OXI. Importantly, the finding of abnormal overnight OXI in this study was reflective of OSA as confirmed in a subset of participants who underwent polysomnography; 91% received a diagnosis of OSA. Additionally, evidence suggests patients with OSA may have refractory hypertension until adequate PAP is initiated (46). To this point, patients triaged to overnight OXI via WHR measurements may avoid lengthy wait times for inpatient polysomnography. In doing so, they could initiate PAP treatment sooner and perhaps reduce the risk of future cardiovascular events. Collectively, our findings support the use of WHR measurements in frontline medical practice due to its technical simplicity, minimal cost, and capacity to predict cardiovascular risk (31), and now, abnormal nocturnal oxygen levels indicative of sleep disordered breathing. Indeed, a joint statement from the American Heart Association, American College of Cardiology, and The Obesity Society reported a limited recommendation for the use of waist circumference in a clinical setting due to a paucity of meta-data (47). The current study builds upon this void and may guide recommendations from these societies which closer align with those of the World Health Organization (30).

### Limitations

Our study is a retrospective cross-sectional analysis with inherent limitations. However, this study is a consecutive series of a relatively large number of participants in a general medical population being seen for screening examinations with OXI, rather than a select population from sleep clinics or patients referred for sleep-related concerns. Our study participants were generally healthy, ambulatory, and exclusively men; thus, our results may not apply to patients with complex problems nor necessarily extend to women. The overall health of participants in this study also makes the pre-test utility of our findings challenging; for instance, previous studies suggest 50% of patients with OSA are hypertensive (48) whereas only 39% of participants have hypertension in the current study. Nevertheless, the clinical significance of abnormal OXI was recently recognized as a predictor of post-operative cardiovascular events in patients undergoing non-cardiac surgical procedures (49). A second limitation is the potentially subjective interpretation of OXI data together with the clinically available information as our end point. However, the pulmonologists who evaluated all available OXI data, including ODI and CT-90, were blinded to the purpose of this manuscript (due to its retrospective nature) and synthesized this information arriving at a specific conclusion. It is the pulmonologist's interpretation of OXI that drives clinical decision making in our practice and likely in other clinical settings as well. An additional consideration is that polysomnography assessments were not rigorously used to follow up on OXI; as such, our ability to determine the rate of false positives or false negatives is attenuated. Nevertheless, in the 88 men who did complete a formal sleep study after abnormal OXI, 91% were found to have OSA.

## Conclusion

In a population of relatively healthy ambulatory middle-aged men, central body fat distribution, as measured with WHR, was strongly associated with the likelihood of an abnormal OXI result. Moreover, our data show measurement of body fat distribution via WHR, a technically simple measurement provides additional predictive value beyond BMI. While our findings need to be confirmed in a prospective study using serial polysomnographic assessments, middle-aged men who snore and have a WHR ≥1.0 should perhaps be directly referred to a sleep clinic or receive cardiorespiratory polygraphy for further evaluation (50). Our study adds to the understanding of central adiposity as it relates to sleep disordered breathing, and how a technically simple measurement, such as the WHR, may assist in screening for, and diagnosing, OSA.

## Data Availability Statement

The raw data supporting the conclusions of this article will be made available by the authors, without undue reservation.

## Ethics Statement

The studies involving human participants were reviewed and approved by Mayo Clinic Institutional Review Board. The patients/participants provided their written informed consent to participate in this study.

## Author Contributions

The study was designed by KR, AC, FL-J, and VS. Statistical analyses of the data were performed by SC and AC. Tables and figures were prepared by JB and KR, as well as JB, KR, KS, and SV contributed to the initial draft of the manuscript. VS is the manuscript's guarantor. All authors critically revised the manuscript, its tables and figures approving it for submission in its present form.

## Funding

This work was supported by Mayo Clinic (KR), the Mayo Clinic Clinician-Investigator Training Program (AC), and the National Institutes of Health (T32HL007111, KS and JB; and HL65176 (SV and VS). The project sponsors were not involved in any stage of study.

## Conflict of Interest

VS has consulted for Baker Tilly, Sleep Number Corporation, Bayer, Jazz Pharmaceuticals and Respicardia. The remaining authors declare that the research was conducted in the absence of any commercial or financial relationships that could be construed as a potential conflict of interest.

## Publisher's Note

All claims expressed in this article are solely those of the authors and do not necessarily represent those of their affiliated organizations, or those of the publisher, the editors and the reviewers. Any product that may be evaluated in this article, or claim that may be made by its manufacturer, is not guaranteed or endorsed by the publisher.
